# Applications of Large Language Models in Glaucoma: A Scoping Review

**DOI:** 10.3390/vision10010009

**Published:** 2026-02-09

**Authors:** Giovanni Rubegni, Alessandra Cartocci, Alessio Luschi, Niccolò Castellino, Francesco Cappellani, Dario Romano, Benedetta Colizzi, Luca Rossetti, Gian Marco Tosi

**Affiliations:** 1 Ophthalmology Unit, Department of Medicine, Surgery and Neurosciences, University of Siena, 53100 Siena, Italy; 2 Department of Medical, Surgical and Neurological Sciences, Dermatology Section, University of Siena, 53100 Siena, Italy; 3 Department of Ophthalmology, University of Catania, 95123 Catania, Italy; 4 Department of Medicine and Surgery, University of Enna “Kore”, Piazza dell’Università, 94100 Enna, Italy; 5 Eye Clinic, ASST Santi Paolo e Carlo, University of Milan, 20142 Milan, Italy

**Keywords:** glaucoma, artificial intelligence, large language models, vision-language models, ChatGPT, clinical decision support, patient education

## Abstract

**Background**: Large language models (LLMs) and vision-language models (VLMs) have recently been applied to ophthalmology for patient education, diagnosis, and surgical decision support. Their ability to generate, interpret, and synthesize medical information positions them as promising assistive tools in glaucoma care. This scoping review aims to consolidate current evidence on the applications of LLMs and VLMSs in glaucoma, summarizing their tasks, inputs, performance metrics, and limitations to guide future clinical and research developments. **Methods**: A systematic search was conducted in PubMed, Scopus, Web of Science, arXiv, and IEEE Xplore from 2014 to July 2025. Eligible studies included original research and research letters employing LLMs or VLMs/MM-LLMs in any glaucoma-related application, including diagnostic reasoning, image interpretation, patient education, or surgical decision support. Screening and full-text review were independently performed by two reviewers following PRISMA-ScR methodology, with discrepancies resolved by consensus. **Results**: In total, 316 records were identified across five databases, with 27 studies meeting the inclusion criteria. The selected studies focused on three main domains: patient education (n = 11), diagnosis and risk prediction (n = 10), and surgical management (n = 6). **Conclusions**: Current LLMs serve best as assistive rather than autonomous tools in glaucoma care. They demonstrate strong potential in patient communication and text-based clinical decision support but remain constrained by variable accuracy, limited multimodal integration, and a lack of ophthalmology-specific fine-tuning. Future research should focus on developing domain-trained and retrieval-augmented LLMs, enhancing multimodal (text-image) fusion, ensuring readability adaptation for patients, and establishing ethical and regulatory frameworks for clinical implementation.

## 1. Introduction

Glaucoma is a chronic, progressive optic neuropathy that, if left untreated, leads to irreversible vision loss and remains one of the leading causes of blindness worldwide [1]. The disease affects more than 70 million people globally, with its prevalence expected to rise due to aging populations and increased life expectancy [2]. Its asymptomatic onset often leads to delayed diagnosis, making early detection and continuous monitoring critical for preserving vision [3]. Traditional diagnostic techniques rely on comprehensive clinical assessments, including intraocular pressure (IOP) measurements, visual field analysis, and optical coherence tomography (OCT) [4]. However, despite advancements in imaging and diagnostic technologies, glaucoma remains underdiagnosed, with a significant proportion of cases detected only at advanced stages, highlighting the need for more effective screening and early detection strategies [5].

The integration of artificial intelligence (AI) into ophthalmology has experienced exponential growth, driven by the increasing availability of large datasets, advanced deep learning architectures, and improvements in computational power [6]. AI-based algorithms have shown high accuracy in disease detection and progression analysis, aiding clinical decision-making [7]. In glaucoma care, AI has shown significant promise in assisting with diagnosis, monitoring disease progression, and predicting the likelihood of future deterioration [8,9].

Recent advances in artificial intelligence have led to the development of large language models (LLMs), deep learning models trained on vast corpora of text to understand, generate, and reason using human-like language. LLMs are capable of performing a wide range of language tasks. In ophthalmology, these models have been applied to patient education, clinical documentation, summarization of scientific literature, and answering disease-related questions [10,11,12]. Among the most widely adopted and influential LLMs are GPT-3/3.5 (OpenAI), LLaMA 1/2/3 (Meta AI), Bard (Google AI), and BERT (Google AI).

Building on these systems, the recent emergence of multimodal large language models (MM-LLMs) marks a new frontier in clinical AI. MM-LLMs, such as GPT-4o (OpenAI) and Gemini 1.5 (Google DeepMind), can simultaneously process and integrate multiple data modalities, including text, images, structured data, and even audio, within a single framework [13]. A prominent subset of MM-LLMs are vision-language models (VLMs), with GPT-4V (OpenAI), Gemini 1.0 (Google DeepMind), Flamingo (DeepMind), and LLaVA (University of Wisconsin–Madison and collaborators) being among the most frequently described and utilized in the recent literature. These models are specifically designed to interpret and generate outputs based on both visual and textual inputs. VLMs typically combine an image encoder with a language model, enabling tasks such as describing clinical images, answering visual diagnostic questions, and synthesizing imaging with narrative clinical data [14,15].

Although still emerging in ophthalmology, MM-LLMs have already shown encouraging results in both clinical and educational applications. In medical education, MM-LLMs have demonstrated strong performance on board-style exam questions and have been used to simulate patient interactions and offer feedback [16,17]. In diagnostic tasks, MM-LLMs have been shown to perform comparably to specialists in identifying diabetic retinopathy [18], keratoconus [19], and keratitis [20]. In therapeutic decision-making, MM-LLMs have shown promising results as a decision support tool in managing diabetic macular edema [21], retinal detachment [22], uveitis [23], ocular oncology [24], and emergency cases [25].

Despite the growing interest in AI-driven approaches to glaucoma care, a dedicated synthesis focusing specifically on large language models (LLMs) and vision-language models (VLMs) in glaucoma is still lacking. Therefore, this scoping review aims to map current glaucoma-specific applications of LLMs/VLMs. Its primary objective is to identify which models have been employed and to classify the specific tasks addressed. The review also examines the types of input data provided to the models (e.g., textual prompts, structured clinical data, and imaging), the methodologies adopted, and the reported outcomes. By mapping the available evidence, this review further highlights key gaps, limitations, and future opportunities for the integration of LLMs/VLMs in glaucoma care.

## 2. Materials and Methods

The findings of this scoping review are reported following the PRISMA (Preferred Reporting Items for Systematic Reviews and Meta-Analyses) extensions for scoping review (PRISMA-ScR) checklist. The protocol for this scoping review was not registered in a public database. The removal of duplicates and the literature screening were carried out using the web-based Rayyan software (Rayyan Systems Inc., Cambridge, MA, USA).

This review includes all studies that employed Large Language Models or Vision Language Models (VLMSs) in glaucoma. Additionally, only original research articles and research letters were included, while preprints and gray literature were excluded to maintain consistency in study quality. Studies published in languages other than English were excluded.

A comprehensive literature search was conducted in PubMed, Scopus, Web of Science, arXiv, and IEEE Xplore, identifying studies published between 2014 and July 2025. The search strategy was designed by researchers (G.R. and A.C.) with documented expertise in the scientific fields relevant to this review.

The search was conducted using titles and abstracts. The specific search queries used for each database are as follows:

- Pubmed: (glaucoma AND (“chatgpt” OR “large language models” OR “LLMs” OR “gemini” OR “bard” OR “gpt” OR “vision language model” OR “BERT” OR “LLaMA” OR “LLaVA” OR “PaLM” OR “foundation model” OR “foundation models” OR “vision-language model” OR “VLMs” OR “CLIP” OR “BLIP” OR “Flamingo”))
- Scopus: TITLE-ABS-KEY (glaucoma AND (“chatgpt” OR “large language model” OR “LLMs” OR “gemini” OR “bard” OR “gpt” OR “vision language model” OR “BERT” OR “LLaMA” OR “LLaVA” OR “PaLM” OR “foundation model” OR “foundation models” OR “vision-language model” OR “VLMs” OR “CLIP” OR “BLIP” OR “Flamingo”))
- Web of Science: TS = (glaucoma AND (“chatgpt” OR “large language model” OR “LLMs” OR “gemini” OR “bard” OR “gpt” OR “vision language model” OR “BERT” OR “LLaMA” OR “LLaVA” OR “PaLM” OR “foundation model” OR “foundation models” OR “vision-language model” OR “VLMs” OR “CLIP” OR “BLIP” OR “Flamingo”))
- arXiv: (glaucoma AND (“chatgpt” OR “large language models” OR “LLMs” OR “gemini” OR “bard” OR “gpt” OR “vision language model” OR “BERT” OR “LLaMA” OR “LLaVA” OR “PaLM” OR “foundation model” OR “foundation models” OR “vision-language model” OR “VLMs” OR “CLIP” OR “BLIP” OR “Flamingo”))
- IEEE Xplore: (glaucoma AND (“chatgpt” OR “large language models” OR “LLMs” OR “gemini” OR “bard” OR “gpt’ OR “vision language model” OR “BERT” OR “LLaMA” OR “LLaVA” OR “PaLM” OR “foundation model” OR “foundation models” OR “vision-language model” OR “VLMs” OR “CLIP” OR “BLIP” OR “Flamingo”))

All articles retrieved from the three databases were compiled in Rayyan, where duplicate entries were removed. The initial screening of the literature was conducted independently and in a blinded manner by two reviewers (G.R. and A.C.), who evaluated only titles and abstracts. Each article was categorized into one of the following groups:

- Included
- Excluded due to incorrect publication type: manuscripts different from the original article (e.g., a review, case series, case report, or abstract) were excluded
- Excluded due to inappropriate study design: The study did not focus on AI applications (LLMs/VLMs) on glaucoma.

Articles that were unanimously classified as included by both reviewers proceeded directly to the full-text screening stage. In cases of discrepant classifications, two additional reviewers (A.L. and D.R.) reassessed the articles and resolved any disagreements.

- During the full-text screening, articles were excluded for the following reasons:
- Inappropriate study design
- Insufficient methodological details (i.e., the methodology lacked essential information, making it impossible to determine the models used).

**Figure 1:**
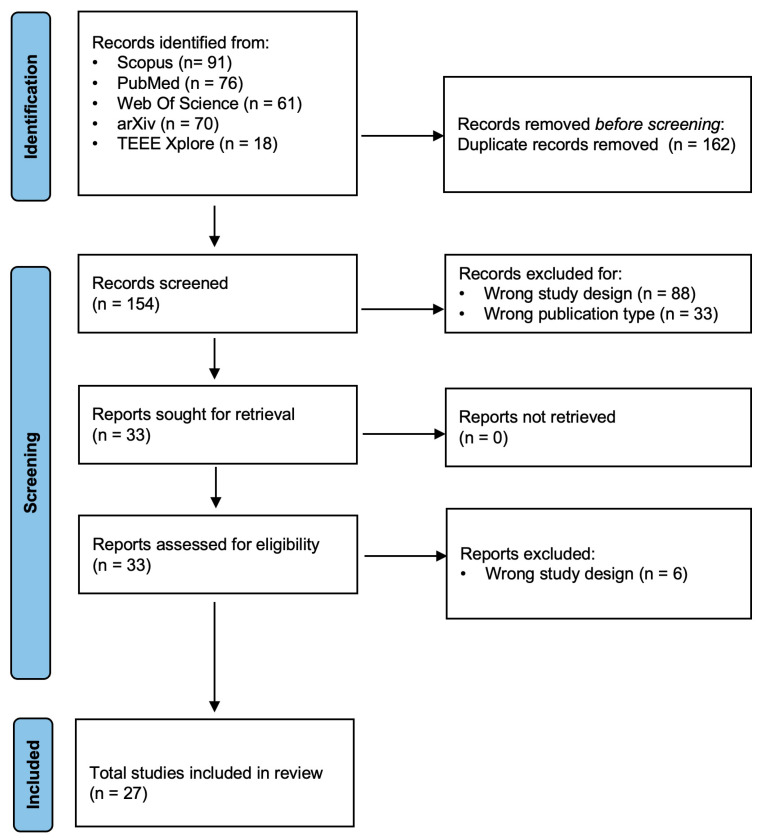
Flow diagram representing the process of selection of the included studies.

Final decisions on article inclusion or exclusion were based on concordant assessments (Figure 1). In cases of continued disagreement, G.T. and L.R. reviewed the full text and reached a resolution through discussion.

## 3. Results

### 3.1. LLMs’ Application in Glaucoma Patient Education

The integration of LLMs, particularly ChatGPT-4, in glaucoma education has been widely explored, with studies assessing their accuracy, readability, and clinical relevance. The following studies focus on the application of LLMs to facilitate comprehension of informational content, respond accurately to patient queries, simplify complex medical concepts, and generate readable educational materials (Table 1).

Kerci et al. [26] found that ChatGPT-4 was satisfactory in responding to general questions regarding glaucoma encountered on the internet; however, in technical, guideline-based questions answered less accurately, indicating shortcomings in its ability to handle expert-level clinical knowledge. Ichhpujani et al. [27] compared the readability and appropriateness of responses given by ChatGPT-3.5 and Google Bard regarding questions related to glaucoma surgery, noting that though ChatGPT-3.5 gave highly accurate responses, its readability score was lower than Bard’s. Tan et al. [28] also showed that ChatGPT-4 achieved a good response accuracy of 70.8% in answering glaucoma questions, and it improved its accuracy when asked to self-correct, indicating potential for iterative learning in patient education. In a different study, Wu et al. [29] found response accuracies of 67% and 61% in answering glaucoma-related questions. However, the researchers observed inconsistency in responses given by LLMs, noting that almost half of ChatGPT-4’s responses changed significantly upon repeated queries. Other studies also compared ChatGPT-4’s ability to generate educational material. Spina et al. [30] and Dihan et al. [31] showed that ChatGPT-4 was able to summarize scientific articles effectively, improving the readability of glaucoma-associated material without loss of semantic coherence compared to expert-authored material. In contrast, Kianian et al. [32] found that ChatGPT-4 struggled to simplify surgical educational material, incapable of decreasing the readability of material regarding glaucoma to a 6th-grade readability, even when asked specifically to do so.

Comparative analyses of LLMs versus existing online educational material have yielded mixed results. Cohen et al. [33] and Mihalache et al. [34] found that ChatGPT-4 was more accurate and comprehensible compared to traditional web-based resources, reinforcing its potential as a reliable patient education tool. Wu et al. [35] compared ChatGPT-4 to Google Assistant and found that both platforms displayed comparable comprehension capabilities; however, neither was ideal for patients of lower literacy levels. In a different study, Wu et al. [36] and Yalla et al. [37] found that ChatGPT-4’s response required a higher reading comprehension capability compared to that of American Academy of Ophthalmology brochures, suggesting that health education outputs of LLMs would be too sophisticated for patients of lower literacy levels. In order to address these challenges, Xue et al. [38] introduced Xiaoqing, a domain-specific large language model for educating patients regarding glaucoma, which was more accurate and readable compared to ChatGPT-4, highlighting the importance of localization in AI-driven health education.

**Table 1:** Summary of the characteristics of the studies about LLMs and glaucoma education.

Reference	Year	Purpose	Input	Model Used	Key Findings
**Kerci et al. [26]**	2024	Evaluating ChatGPT-4’s accuracy on web-based and guideline-based glaucoma questions, using glaucoma specialist responses as the reference standard	Textual patient questions from the internet	ChatGPT-4	88.7% accuracy in web-based questions, 75% in guideline-based questions
**Ichhpujani et al. [27]**	2024	Comparing ChatGPT-3.5 and Google Bard in answering glaucoma surgery questions, using glaucoma specialist responses as the reference standard	Textual questions about glaucoma surgery	ChatGPT-3.5, Google Bard	ChatGPT-3.5 was highly accurate (96% vs. 68%) but had lower readability than Bard
**Tan et al. [28]**	2024	Assessing ChatGPT-4’s accuracy on glaucoma questions and its ability to self-correct, answers assessed by glaucoma specialists	Textual glaucoma-related questions	ChatGPT-4	Accuracy improved from 30.6% to 57.1% after self-correction
**Wu et al. [29]**	2022	Analyzing ChatGPT-4’s response accuracy and consistency in repeated glaucoma-related queries, answers assessed by glaucoma specialists	Textual glaucoma-related questions	ChatGPT-4	Accuracy was 67% and 61% on two occasions; 48% of responses changed significantly
**Spina et al. [30]**	2025	Assessing ChatGPT-4’s ability to simplify glaucoma educational material	Textual scientific articles on glaucoma	ChatGPT-4	Readability improved by 30% (FKGL reduction); maintained high semantic consistency (cosine similarity = 0.861)
**Dihan et al. [31]**	2024	Comparing ChatGPT-3.5, ChatGPT-4, and Bard in generating childhood glaucoma education content	Textual scientific articles on glaucoma	ChatGPT-3.5, ChatGPT-4, Bard	All models produced high-quality, accurate educational materials; ChatGPT-4 had the best readability (FKGL = 4.8)
**Kianian et al. [32]**	2023	Assessing ChatGPT-4’s ability to simplify existing online educational content on glaucoma surgical procedures to a 6th-grade readability level	Textual glaucoma educational material	ChatGPT-4	ChatGPT-4 failed to reduce readability level (FKGL = 11 vs. 12, *p* = 0.34) compared to online edcuational webpages
**Cohen et al. [33]**	2024	Comparing ChatGPT-4 to traditional web-based educational materials in glaucoma FAQ, answers assessed by glaucoma specialists	Textual glaucoma-related questions	ChatGPT-4	ChatGPT-4 had higher accuracy (97% vs. 77%) but required a higher reading level (14.3 vs. 9.4) than web-based educational material
**Mihalache et al. [34]**	2024	Analyzing ChatGPT-3.5 and 4’s accuracy and readability in answering glaucoma questions, answers assessed by expert ophthalmologists	Textual glaucoma-related questions	ChatGPT-3.5, ChatGPT-4	GPT-4 outperformed GPT-3.5 (median score 5 vs. 3) on accuracy by expert grading; GPT-4 responses were longer in duration and word count
**Wu et al. [35]**	2023	Comparing ChatGPT-4 and Google Assistant in simplifying glaucoma-related queries, with responses comprehension assessed by expert ophthalmologists	Textual glaucoma-related questions	ChatGPT-4, Google Assistant	ChatGPT-4 and Google Assistant had similar comprehension (9.5–13.4 vs. 9.6–11.3 education years)
**Wu et al. [36]**	2024	Comparing the readability of ChatGPT-4-generated responses and AAO brochures in answering glaucoma-related questions	Textual glaucoma educational material	ChatGPT-4	ChatGPT-4 responses required grade 12.5 comprehension vs. AAO materials (grade 9.4, *p* = 0.0384)
**Yalla et al. [37]**	2024	Comparing ChatGPT-4, Bard, Bing, and AAO educational material in answering glaucoma-related questions	Textual glaucoma-related questions	ChatGPT-4, Bard, Bing	ChatGPT-4 had the highest accuracy (4.26/5) but the worst readability (FKGL = 13.01) compared to Bard and Bing
**Xue et al. [38]**	2024	Comparing Xiaoqing, a domain-specific LLMS, to ChatGPT-4 in its ability to generate accurate and readable glaucoma education responses, as evaluated by an expert ophthalmologist, answers were assessed by expert ophthalmologists	Textual glaucoma-related questions	Xiaoqing, ChatGPT-4	Xiaoqing outperformed ChatGPT-4 in accuracy (92.7% vs. 87.5%) and readability (FKGL = 8.4 vs. 10.1)

Legend: FKGL: Flesch-Kincaid Grade Level; AAO: American Academy of Ophthalmology.

### 3.2. LLMs’ Application in Glaucoma Diagnosis

The role of LLMs in diagnosing glaucoma has been widely explored, specifically their potential to process clinical data, interpret imaging reports, predict disease progression, assist in differential diagnosis, and support decision-making in the clinic (Table 2).

Mihalache et al. [39] found that while ChatGPT-4 performed well in answering diagnostic questions, its ability to interpret ophthalmic images remained limited, performing better in text-based scenarios compared to those that used imaging.

Beyond their use in diagnosis, LLMs have been investigated for their potential in glaucoma risk prediction. Choi et al. [40] presented that ChatGPT-4 was accurate in identifying high-risk patients, showing performance comparable to traditional machine learning models. Similarly, Huang et al. [41] found that LLMs could be useful in early glaucoma screening, as ChatGPT-4 accurately predicted progression from ocular hypertension to glaucoma, supporting its integration into risk assessment programs.

Comparisons between human ophthalmologists and LLMs have shown different results. Delsoz et al. [42] established that ChatGPT-4’s diagnostic capability in case reports was on par with that of senior ophthalmology residents, suggesting potential utility in triaging patients with glaucoma and supporting decision-making processes. Zhang et al. [43] established that GPT-4o performed poorly in the primary diagnosis of glaucoma cases compared to human ophthalmologists, though it was highly accurate in differential diagnosis, suggesting a need for AI-assisted processes rather than complete AI-independence.

Finally, recent studies assessed LLMs’ capabilities in glaucoma image-based diagnosis. AlRyalat et al. [44] and Jalili et al. [45] evaluated multimodal LLMs in fundus image diagnosis, indicating that while these models showed high accuracy in glaucoma diagnosis, consistency across datasets remained a challenge, limiting their current applicability in real-world settings. Raja et al. [46] compared ChatGPT versions 3.5 and 4.0’s diagnostic performance using clinical and anamnestic information combined with optic head fundus imaging. ChatGPT 4.0 showed accuracy and specificity above 85%, better than ChatGPT 3.5, even if ChatGPT 3.5 was found to be more sensitive. Advancements in LLM-integrated ensemble deep learning models have further enhanced diagnostic accuracy. Wang et al. [47] created an artificial intelligence system that combined retinal fundus images, vascular segmentation, and region of interest analysis, demonstrating significant improvements in detection rates compared to conventional single-image analysis. More sophisticated systems have recently been developed, combining the text generation capabilities of LLMs with vision-language models to enhance clinical accuracy.

Bae et al. [48] proposed a novel approach combining fundus images with textual descriptions generated by GPT-3 to enhance diagnostic accuracy through a vision-language model (the CLIP model). Their findings indicated that incorporating LLMs-generated optic disk descriptions enhanced the performance of the CLIP model, achieving an accuracy of 0.89 in distinguishing glaucomatous from non-glaucomatous optic disks based on fundus images.

Vishwakarma et al. [49] propose a multi-modal glaucoma detection framework that combines transfer learning, explainable AI (LIME), and LLMs to enhance both the accuracy and interpretability of glaucoma diagnosis from fundus images. By combining a VGG16-based vision model with LLMs-generated descriptions of the optic disk, the model reached an accuracy of 80.19% in identifying glaucomatous optic disks.

**Table 2:** Summary of the characteristics of the studies about LLMs and glaucoma diagnosis.

Reference	Year	Purpose	Input	Model Used	Key Findings
**Mihalache et al. [39]**	2024	Evaluating ChatGPT-4’s ability to interpret fundus images for glaucoma diagnosis	Fundus images	ChatGPT-4	Accuracy: 70% across 429 questions; performed better on non-image questions (82%) vs. image-based (65%)
**Delsoz et al. [42]**	2024	Evaluating ChatGPT’s diagnostic ability in identifying glaucoma subtypes and non-glaucomatous conditions using clinical case reports, compared to that of ophthalmology residents	Textual patient clinical data	ChatGPT-4	GPT’s correct diagnosis in 8/11 cases (72.7%); ophthalmology residents scored between 54.5 and 72.7%
**Choi et al. [40]**	2025	Developing a glaucoma risk prediction model using demographic, clinical, and laboratory factors	Textual patient structured clinical data	ChatGPT-4	Predictive ability is comparable to traditional machine learning. Glaucoma OR: 1.87 (low risk), 2.72 (moderate risk), 15.36 (high risk)
**Zhang et al. [43]**	2024	Comparing the diagnostic performance of GPT-4o with human ophthalmologists in glaucoma diagnosis from clinical case reports	Textual patient clinical data	ChatGPT-4	GPT-4 performed worse in primary diagnosis than the best ophthalmologist (score: 5.5 vs. 8.0), but comparable in differential diagnosis (7.58 vs. 7.61)
**Raja et al. [46]**	2025	Assessing ChatGPT-3.5 and ChatGPT-4.0 in diagnosing glaucoma using anamnestic data and fundus images from a public dataset	Textual patient clinical data and fundus images	ChatGPT-3.5, ChatGPT-4.0	ChatGPT-4.0 accuracy: 87%; specificity = 90%; sensitivity = 61%. ChatGPT-3.5 was more sensitive (85%) but had lower accuracy (66%)
**Jalili et al. [45]**	2025	Evaluating GPT-4V’s ability to identify glaucoma-relevant features, such as cup-to-disk ratio, from fundus images	Fundus images	ChatGPT-4	GPT-4V accuracy varied: 0.68–0.81; lower agreement with expert graders (Cohen kappa: 0.08–0.72); consistent in assessing cup-to-disk ratio
**AlRyalat et al. [44]**	2024	Assessing ChatGPT-4’s ability to classify fundus images as glaucomatous or non-glaucomatous	Fundus images	ChatGPT-4	ChatGPT-4 accuracy: 90%; specificity: 94.44%; sensitivity: 50%
**Huang et al. [41]**	2024	Investigating ChatGPT’s capability to predict conversion from ocular hypertension to glaucoma using structured clinical variables	Structured clinical data (IOP, CCT, CDR, Visual field MD, and PSD)	ChatGPT-4	ChatGPT-4.0 accuracy: 75%; sensitivity: 56%; specificity: 78%. ChatGPT-3.5 accuracy: 61%
**Wang et al. [47]**	2024	Developing an ensemble deep learning model based on VLM to classify glaucoma status from fundus images	Fundus images (with vessel segmentation and region of interest analysis)	VLM-based ensemble model	Ensemble model achieved: 98.7% accuracy; sensitivity: 90%; specificity: 99.7% (REFUGE dataset). Accuracy: 83%; sensitivity: 76.4%; specificity: 85.4% (ORIGA dataset); Accuracy: 98.5%; sensitivity: 98.3%; specificity: 98.6% (G1020 dataset)
**Bae et al. [48]**	2024	Evaluating glaucoma classification accuracy by using LLM-generated optic disk descriptions as input for a CLIP-based vision-language model	Fundus images with generated textual descriptions of the optic disk	LLM-augmented CLIP model	CLIP baseline accuracy: 80.3%; CLIP + ChatGPT accuracy: 89.0%; CLIP + GPT-4V accuracy: 87.3%; Ensemble (ChatGPT + GPT-4V) accuracy: 88.5%
**Vishwakarma et al. [49]**	2025	Developing a multimodal AI system integrating LLM-generated optic disk image descriptions and a deep learning model to classify glaucomatous optic disks	Fundus images with generated textual descriptions of the optic disk	Multi-modal deep learning + LLM framework	Deep learning models achieved: VGG16 (accuracy: 80.19%, AUC: 0.89), ResNet50 (accuracy: 79.81%, AUC: 0.88), VGG19 (accuracy: 78.85%, AUC: 0.87), InceptionV3 (accuracy: 78.46%, AUC: 0.85), and DenseNet121 (accuracy: 76.92%, AUC: 0.84)

Legend: OR: Odds Ratio; IOP: intraocular pressure; CCT: central corneal thickness; CDR: cup to disk ratio; AUC: area under the curve.

### 3.3. LLMs in Glaucoma Surgical Management

Few studies have demonstrated LLMs’ potential in glaucoma surgical evaluation and management, analyzing LLMs’ ability to provide surgical recommendations, classify postoperative complications, predict the need for surgical intervention, and support decision-making in complex surgical cases through the analysis of structured and unstructured clinical data (Table 3).

Carlà et al. [50] investigated ChatGPT-4 and Google Gemini’s capability to review in-depth case descriptions of glaucoma to generate appropriate surgical plans. ChatGPT-4 demonstrated significantly higher accuracy in surgical decision-making, agreeing with expert advice in 58% of cases compared to 32% with Google Gemini. In challenging surgical scenarios, ChatGPT-4 maintained good agreement with specialists, whereas Gemini performed considerably worse, often failing to complete the task. This study highlights ChatGPT-4’s potential as a decision support system for ophthalmologists, especially in complex situations that require advanced reasoning.

Besides surgical planning, LLMs demonstrated potential in glaucoma surgical complications management. Shaheen et al. [51] evaluated ChatGPT’s potential to classify postoperative hemorrhagic events (HEs) after microinvasive glaucoma surgery (MIGS) using electronic health record (EHR) data. Their study found that ChatGPT was highly accurate in classifying hemorrhagic events, supporting its potential role in automated EHR analysis for postoperative monitoring. Such results indicate that LLMs can be useful tools in monitoring postoperative complications and predictive analysis.

Hu et al. [52] explored the predictive power of transformer-based LLMs, including BERT and RoBERTa, using free-text clinical notes from electronic health records. Fine-tuned models were able to predict which glaucoma patients would require surgery with an accuracy of up to 83%. This study demonstrated the potential of LLMs to extract prognostic insights from unstructured text, even outperforming manual clinician review in predictive accuracy.

**Table 3:** Summary of the characteristics of the studies about LLMs and glaucoma surgical management.

Reference	Year	Purpose	Input	Model Used	Key Findings
**Carlà et al. [50]**	2024	Assessing ChatGPT-4 and Google Gemini’s ability to generate appropriate surgical recommendations for glaucoma, as compared with expert ophthalmologists’ decisions	Textual patient clinical data	ChatGPT-4, Google Gemini	ChatGPT-4 agreed with expert surgical plans in 58% of cases vs. 32% for Google Gemini; performed better in complex cases requiring advanced reasoning
**Shaheen et al. [51]**	2025	Assessing ChatGPT’s ability to classify postoperative hemorrhagic events after MIGS, using expert clinician annotations as a reference standard	Textual patient clinical data	ChatGPT-4	ChatGPT was highly accurate (AUC = 0.985) in classifying hemorrhagic events
**Hu et al. [52]**	2024	Evaluating transformer-based LLMs for predicting glaucoma progression requiring surgery from unstructured ophthalmology clinical notes	Textual patient clinical data	BERT, RoBERTa, BioBERT, DistilBERT	RoBERTa model achieved an accuracy of 0.83 in predicting glaucoma progression to surgery, outperforming the standard BERT model (Accuracy = 0.76)

Legend: MIGS: minimally invasive glaucoma surgery; AUC: area under the curve.

## 4. Discussion

The development of Large Language Models for glaucoma presents both significant challenges and opportunities.

### 4.1. Challenges in the Application of LLMs in Glaucoma

Despite their potential in managing glaucoma, various challenges limit their application in clinical settings and practical use:

1. Limited Performance on Technical and Guideline-Based Queries: Though LLMs accurately answer basic questions related to glaucoma, their accuracy decreases when dealing with complex, guideline-oriented questions. Their inability to apply deep clinical reasoning and interpret specialized ophthalmic terminology restricts their use in providing accurate, evidence-based advice to healthcare providers [53].
2. Inconsistency and Content Stability: It has been established that responses given by LLMs differ when asked repeatedly, raising concerns about content consistency in patient education and clinical decision-making. This instability challenges their use in standardized medical reporting, where consistency in recommendations is crucial [54].
3. Limited Capability in Image-Based Diagnosis: While LLMs demonstrate strong textual analysis skills, their ability to interpret ophthalmic imaging, including fundus photographs and OCT scans, remains inadequate. Even multimodal LLMs that combine vision and text analysis exhibit inconsistency in their diagnostic capabilities, rendering them unreliable for independent diagnosis of glaucoma. These limitations stem from several factors, including insufficient training on large, high-quality ophthalmic image datasets and variability in imaging protocols across devices and institutions [55,56].
4. Readability and Accessibility Issues: Educational material developed using LLMs is often of a higher comprehension level compared to regular patient educational material, making it more accessible to individuals with low health literacy. Though LLMs can simplify content, they struggle to effectively balance readability with medical accuracy, especially when detailing surgical procedures and complex treatment options [57].
5. Challenges in Surgical Decision-Making and Postoperative Care: Though LLMs have been investigated for use in glaucoma surgical planning, their precision in making complex decisions remains mediocre. They can provide general recommendations yet fail to adapt to patient-specific variables or intraoperative observations. Moreover, though LLMs can classify postoperative complications, they do not yet use real-time clinical observations to inform dynamic postoperative management [58].
6. Variability in Health Systems and Clinical Protocols: The diverse medical data utilized to train for LLMs means that it is hard to normalize their response across various healthcare systems. The variability in patterns of treatment in various settings, surgeries, and procedures limits their application in every setting, making it a requirement to be localized and tuned to specific groups of patients [59].
7. Regulatory and Ethical Hurdles: The black-box nature of LLMs, in conjunction with concerns of bias, misinformation, and legal accountability, is a key barrier to their application in clinical ophthalmology. The lack of transparent decision-making processes and uncertainty regarding medical liability are challenges to healthcare institutions adopting LLM-based models in the treatment of patients [60].

### 4.2. Future Directions to Develop LLMs in Glaucoma

For their full inclusion in glaucoma clinical and surgical workflow, future studies and development should work towards overcoming their existing shortcomings:

1. Enhancing Performance on Technical and Guideline-Based Queries: Training LLMs on structured, domain-specific datasets curated from peer-reviewed ophthalmology literature and clinical guidelines could enhance understanding of specialist medical vocabulary. The application of reinforcement learning with human feedback by glaucoma specialists could refine their ability to correctly interpret and execute evidence-based procedures [61].
2. Improving Consistency and Content Stability: Application of retrieval-augmented generation (RAG), in which LLMs retrieve answers straight from verified medical repositories, could help ensure response consistency across multiple queries. The application of finely tuned models having self-validation mechanisms can also reduce answer variability and misinformation, increasing the reliability of LLMs in generating glaucoma educational material and clinical guidance. Additionally, consistency and content stability can be further improved through careful adjustment of generation parameters and prompt engineering, which help guide the model toward producing more uniform and context-appropriate outputs [62].
3. Advancing Multimodal Capabilities for Image-Based Diagnosis: LLMs with deep learning vision models trained on OCT scans, fundus images, and visual fields would enhance AI-assisted diagnostic accuracy. Next-generation systems would require fusion models that combine imaging and text-based input to enable a comprehensive, AI-assisted approach to glaucoma detection and progression analysis [63].
4. Optimizing Readability and Accessibility of AI-Generated Educational Content: Enabling LLMs to dynamically adapt to patients’ reading levels may improve the accessibility of AI-generated educational materials. Several studies included in this review assessed readability using standardized metrics such as the Flesch Reading Ease Score, the SMOG Index, and the Flesch-Kincaid Grade Level, which provide objective measures to compare the comprehensibility of LLMs’ outputs [64]. Incorporating these metrics into the development and evaluation of LLM-generated content can help ensure that materials are suitable for diverse patient populations. Additionally, interactive, dialog-based models that allow patients to ask clarifying questions and the incorporation of visual aids (e.g., infographics or videos) may further enhance comprehension while preserving medical accuracy [65].
5. Refining LLM-Assisted Surgical Decision-Making and Postoperative Monitoring: developing LLMs to integrate live input of patients’ electronic health records in real time would enable personalized surgical recommendations and postoperative complication management. However, current limitations, such as variability in EHR formats, data privacy concerns, and the models’ limited ability to interpret dynamic clinical contexts, must be addressed. AI-assisted decision support systems would need to be developed to augment, not replace, clinician knowledge and allow AI-generated surgical insights to remain contextually relevant and adaptable to individual cases [66].
6. Standardizing LLMs for Global Healthcare Applications: To address variabilities in clinical guidelines, future AI models should be customized for different healthcare systems by incorporating region-specific treatment protocols. AI developers, regulatory institutions, and healthcare institutions would need to collaborate to enable AI-assigned recommendations to be compatible across different global healthcare practices [67].
7. Establishing Ethical and Regulatory Frameworks for LLMs Deployment in Ophthalmology: developing transparent, explainable AI (XAI) models would help increase clinician trust and facilitate regulatory approval. Additionally, implementing clear liability guidelines for AI-assisted recommendations and enforcing strict requirements for models’ certification in a clinical setup would be crucial for safe and ethical integration into ophthalmic care [68].

## 5. Conclusions

Large Language Models offer promising applications in glaucoma diagnosis, management, and surgical decision-making but face challenges in their clinical adoption due to concerns related to accuracy, consistency, image-based diagnostics, readability, and regulatory compliance.

Taken together, the evidence mapped in this scoping review suggests that LLMs currently perform most reliably in glaucoma when the task is predominantly text-based and supportive rather than autonomous and diagnostic. Their strengths are most apparent in patient education and communication-oriented workflows, including drafting or refining educational material, answering common patient questions, summarizing glaucoma-related information, and supporting documentation—use cases in which outputs can be standardized, checked for accuracy, and adjusted for readability by clinicians. Conversely, performance becomes less dependable as tasks move toward high-stakes clinical decision-making, particularly when responses must reflect guideline-level precision, remain stable across repeated prompts, or incorporate image-based interpretation (e.g., fundus photographs or OCT), where variability in imaging quality and datasets can undermine consistency and generalizability. Similarly, in surgical planning and complex management scenarios, LLMs may assist clinicians by organizing narratives and highlighting relevant factors, but current agreement with expert decisions remains insufficient for unsupervised use. Therefore, the most appropriate near-term role of LLMs/VLMs in glaucoma care is as clinician-supervised assistive tools that enhance education, workflow efficiency, and structured risk communication, while diagnostic and therapeutic recommendations, especially those derived from images, should be considered adjunctive at most, requiring local validation, explicit uncertainty communication, and clear governance to minimize safety and liability risks.

Technological advancements in structured training set generation and reinforcement learning would improve LLMs’ performance in answering complicated, guideline-aligned questions to provide evidence-based advice to healthcare professionals. Integration with deep learning vision models and electronic health records (EHRs) will play an essential role in harmonizing text analysis with imaging evaluation to ultimately enhance disease detection and personalized treatments.

Additionally, LLMs’ adaptation to align with regional healthcare standards and establishing clear ethical and regulatory frameworks will be critical for their safe adoption in ophthalmology.

This scoping review has limitations related to the review process itself. First, in line with PRISMA-ScR guidance, we did not perform a formal risk-of-bias or methodological quality assessment, which limits our ability to comment on the internal validity of individual studies and the certainty of their estimates. Second, we restricted inclusion to English-language publications and excluded gray literature; together with potential publication bias (i.e., preferential publication of positive findings), this may have influenced the evidence base captured. In addition, the included evidence was heavily skewed toward GPT-family models (85% of the included studies), which limits the generalizability of the mapped findings to other model families. Finally, the included studies were predominantly conducted in high-income countries, which may limit global generalizability to settings with different languages, health literacy levels, resource availability, clinical workflows, and imaging devices.

## Data Availability

No new data were created or analyzed in this study.
